# Supplementary material for: PEGylated Strontium Sulfite Nanoparticles with Spontaneously Formed Surface-Embedded Protein Corona Restrict Off-Target Distribution and Accelerate Breast Tumour-Selective Delivery of siRNA
Source: J Funct Biomater. 2022 Nov 1;13(4):211. doi: 10.3390/jfb13040211 (PMC9680366; doi:10.3390/jfb13040211)
Supplement: Supplementary file 1 [file jfb-13-00211-s001.zip › jfb-1930106-supplementary.pdf]

Supplementary Material:

PEGylated Strontium Sulfite Nanoparticles with Spontaneously Formed Surface-Embedded Protein Corona Restrict Off-Target Distribution and Accelerate Breast Tumour-Selective Delivery of siRNA

Md. Emranul Karim and Ezharul Hoque Chowdhury *

Jeffrey Cheah School of Medicine and Health Sciences, Monash University Malaysia, Jalan Lagoon Selatan, Bandar Sunway, Petaling Jaya 47500, Malaysia
* Correspondence: md.ezharul.hoque@monash.edu; Tel.: +60-3-5514-4978; Fax: +60-3-5514-6323

Table S1. Identification, characterization and functions of the proteins found with SSNs following incubation with 10% of mice plasma.

Protein classes	Identified proteins	-10lgP	Coverage	Mass	Functions
Transport proteins	Albumin 1	156.86	31	68693	chaperone binding, DNA binding, fatty acid binding, identical protein binding, oxygen binding, pyridoxal phosphate binding and toxic substance binding
Enzymes	Glutamine synthetase	38.65	4	42019	glutamine biosynthetic process
Structural proteins	Keratin 16	32.67	1	51606	structural constituent of cytoskeleton
Structural proteins	Keratin 16	32.67	1	51693	structural molecule activity
Structural proteins	Keratin intermediate filament 16b	32.67	1	51966	structural molecule activity
Structural proteins	Keratin intermediate filament 16a	32.67	1	52053	structural molecule activity
Structural proteins	Uncharacterized protein	24.55	2	33882	structural molecule activity
Structural proteins	Keratin 24 variant 2	24.55	2	40994	structural molecule activity
Structural proteins	Keratin 19	24.55	2	44542	protein-containing complex binding, structural constituent of muscle
Structural proteins	Keratin, type I cuticular Ha2	24.55	2	51153	structural molecule activity
Structural proteins	Keratin 15, isoform CRA_a	24.55	2	49494	scaffold protein binding, structural molecule activity
Structural proteins	Keratin, type I cytoskeletal 10	24.55	1	57041	protein heterodimerization activity, structural constituent of epidermis
Structural proteins	Nup205	23.83	1	69494	structural constituent of nuclear pore
Transport Proteins	Conserved oligomeric Golgi complex subunit 7	23.83	1	80582	intracellular protein transport
Transport Proteins	Conserved oligomeric Golgi complex subunit 7	23.83	1	86075	intracellular protein transport
Enzymes	Ercc5 protein	22.76	1	86901	endonuclease activity, single-stranded DNA binding
Enzymes	Nek1 protein	21.82	2	48636	ATP binding, protein serine/threonine kinase activity
Enzymes	Nek1 protein	21.82	1	133856	ATP binding, protein kinase activity

Enzymes	Nek1 protein	21.82	1	139659	ATP binding, protein kinase activity
Enzymes	MKIAA1901 protein	21.82	1	139947	ATP binding, protein kinase activity
Enzymes	Nek1 protein	21.82	1	144269	ATP binding, protein kinase activity
others	WD repeat-containing protein 81	21.82	1	211931	mitochondrion organization

Table S2. Identification, characterization and functions of the proteins found with PEG-SSNs following incubation with 10% of mice plasma.

Protein classes	Identified protein	-10lgP	Coverage (%)	Mass	Function
Structural proteins	Keratin, type I cytoskeletal 10	160.85	39	57041	Protein heterodimerization activity, structural constituent of the epidermis.
Structural proteins	Keratin 77	134.13	15	61359	structural molecule activity
Structural proteins	Keratin 77	134.13	15	61302	structural molecule activity
Transport proteins	Albumin 1	131.44	16	68693	chaperone binding, DNA binding, fatty acid binding, identical protein binding, oxygen binding, pyridoxal phosphate binding and toxic substance binding
Structural proteins	Keratin, type II cytoskeletal 6B	126.58	11	59526	structural molecule activity
Structural proteins	Krt6b protein	126.58	11	60191	structural molecule activity
Structural proteins	Krt6b protein	126.58	11	60273	structural molecule activity
Structural proteins	Keratin 5	112.68	12	61767	scaffold protein binding, structural molecule activity
Structural proteins	Krt2 protein	99.11	11	70923	structural molecule activity
Structural proteins	Keratin 15, isoform CRA_a	95.8	10	49494	scaffold protein binding, structural molecule activity
Structural proteins	Keratin Kb40	92.26	2	85239	structural molecule activity
Structural proteins	Keratin 78	92.26	1	112265	structural molecule activity
Structural proteins	Krt78 protein	92.26	3	54765	structural molecule activity
Structural proteins	Krt78 protein	92.26	3	54774	structural molecule activity
Structural proteins	Krt78 protein	92.26	3	56780	structural molecule activity
Structural proteins	Type II cytokeratin Kb40	92.26	3	47619	structural molecule activity

Structural proteins	Uncharacterized protein	81.33	5	58266	structural molecule activity
Structural proteins	Uncharacterized protein	81.33	5	58240	structural molecule activity
Structural proteins	Keratin 90	81.33	5	58224	structural molecule activity
Structural proteins	Keratin 16	69	5	51606	structural molecule activity
Structural proteins	Keratin 16	69	5	51693	structural molecule activity
Structural proteins	Keratin intermediate filament 16b	69	5	51966	structural molecule activity
Structural proteins	Keratin intermediate filament 16a	69	5	52053	structural molecule activity
